# Radiation damping strongly perturbs remote resonances in the presence of homonuclear mixing

**DOI:** 10.5194/mr-3-43-2022

**Published:** 2022-02-24

**Authors:** Philippe Pelupessy

**Affiliations:** Laboratoire des Biomolécules (LBM), Département de Chimie, École Normale Supérieure, PSL University, Sorbonne Université, CNRS, 75005 Paris, France

## Abstract

In this work, it is experimentally shown that the weak oscillating magnetic field (known as the “radiation damping” field) caused by the inductive coupling between the transverse magnetization of nuclei and the radio frequency circuit perturbs remote resonances when homonuclear total correlation mixing is applied. Numerical simulations are used to rationalize this effect.

## 1 Introduction

The inductive coupling between precessing magnetization and a radio frequency (RF) circuit creates an RF field, which, in turn, affects the evolution of the magnetization and, hence, the appearance of nuclear magnetic resonance (NMR) spectra. The existence of this phenomenon was first hypothesized by [Bibr bib1.bibx27], and a more rigorous theoretical description was later provided by [Bibr bib1.bibx4]. The latter work introduced the term “radiation damping” (RD), an expression which, as several authors have previously stated ([Bibr bib1.bibx1]), is rather misleading, with both radiation and damping being called into question. The expression “radiation feedback” has been suggested as an alternative; however, this term is often used to designate active feedback circuits to enhance ([Bibr bib1.bibx28]) or eliminate ([Bibr bib1.bibx17]) the effects of radiation damping. Another option, in analogy with quantum back action, could be “induction back action”. In order to avoid confusion, the term RD will, nevertheless, be employed in this work. When no other RF fields are present, the RD field rotates the magnetization that is responsible for the induced RF field towards its equilibrium direction ([Bibr bib1.bibx5]), parallel to the main field, leaving the norm unchanged (if it is homogeneous in space). In liquid-state NMR, this effect is usually weak and only noticeable when the magnetization is strong, either for nuclei in molar concentrations with high gyromagnetic ratios (in particular, solvents containing hydrogen) or when the polarization is enhanced. It increases with higher quality factors Q . The RD field strongly affects the resonances with frequencies close to the one that is at its origin ([Bibr bib1.bibx22]) and remote resonances that are directly coupled by scalar or dipolar interactions ([Bibr bib1.bibx19]) or undergo chemical exchange ([Bibr bib1.bibx8]) with the nuclei that induce RD. Subtle effects on remote resonances ([Bibr bib1.bibx26]) can affect sensitive difference experiments. Homonuclear isotropic mixing sequences which have been designed for total correlation spectroscopy (TOCSY), however, are very efficient at removing the chemical shift differences from the effective Hamiltonian ([Bibr bib1.bibx6]). In this work, it will be shown that RD, in the presence of suitable mixing sequences, can heavily perturb spins over a wide range of resonance frequencies.

**Figure 1 Ch1.F1:**
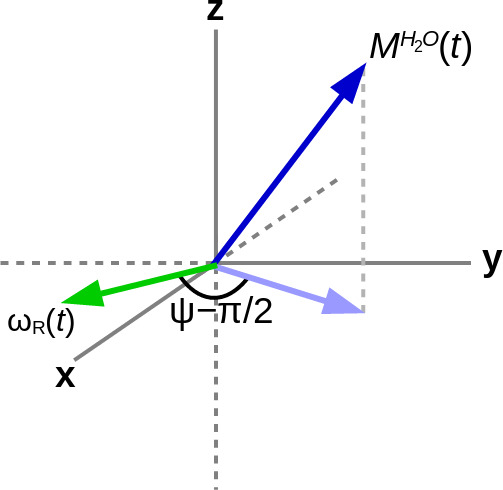
The RD field $\omega_{R}(t)$ (green arrow) lies in the xy plane. It has an amplitude that is proportional to the projection (light-blue arrow) of the water magnetization $M^{H_{2}O}(t)$ (dark-blue arrow) onto the same plane and a phase of ψ-π/2 with respect to this projection.

## 2 Materials and methods

All experiments have been performed on a Bruker NMR spectrometer in a field of 14.1 T (600 MHz proton frequency) equipped with a probe cooled by liquid nitrogen (“Prodigy”) with coils to generate pulsed field gradients along the z axis. This study has been done on a standard calibration sample that contained, among other substances, about 80 % H ${}_{2}$ O and 20 % HDO (i.e., close to 100 M solvent protons) and 0.5 mM sodium 3-(trimethylsilyl)propane-1-sulfonate (DSS). At the experimental temperature of 298 K, the chemical shift difference between the solvent and the methyl protons is ca. 4.78 ppm (2868 Hz at 14.1 T, the water resonance being “downfield”, i.e., precessing at a higher negative frequency).

The variants of the selective TOCSY experiment ([Bibr bib1.bibx10]) used in this work, with an optional bipolar gradient pair for coherence pathway selection ([Bibr bib1.bibx9]), are described in Fig. [Fig Ch1.F2]. A selective pulse applied to the solvent A , followed by a pulsed field gradient, can be inserted before the sequence so that the magnitude of the longitudinal magnetization $M_{z}^{A}$ can be controlled and, hence, the strength of the RD effect. If, for example, the pulse rotates the magnetization into the xy plane, RD should play no role in the remainder of the sequence, except if the dephased magnetization is (accidentally) refocused. If, instead of the transverse magnetization, one wishes to monitor the z component of the magnetization that remains after the homonuclear mixing sequence, a gradient followed by a π/2 pulse can be inserted just before acquisition. For homonuclear transfer, an isotropic mixing pulse train, DIPSI-2 (Decoupling In the Presence of Scalar Interactions; [Bibr bib1.bibx21]), has been chosen with an RF amplitude $\gamma B_{1}/2\pi =4.17$ kHz (which corresponds to a duration of 60 µs for a π/2 pulse). Selective excitation, either on the water or on the methyl protons, has been achieved with a Gaussian π/2 pulse of 5 ms. The programs for the numerical simulation of the trajectories of the magnetization (see the Supplement for the code) and to extract the experimental peak intensities were written in the Python language. In particular, the evolution of the magnetization under the DIPSI-2 pulse train (governed by the set of nonlinear coupled differential Eqs. [Disp-formula Ch1.E1]–[Disp-formula Ch1.E3]) was numerically evaluated with the SciPy integration libraries ([Bibr bib1.bibx30]) using an explicit Runge–Kutta method of order 5 (RK5(4)) ([Bibr bib1.bibx23]).

## 3 Experimental results

**Figure 2 Ch1.F2:**
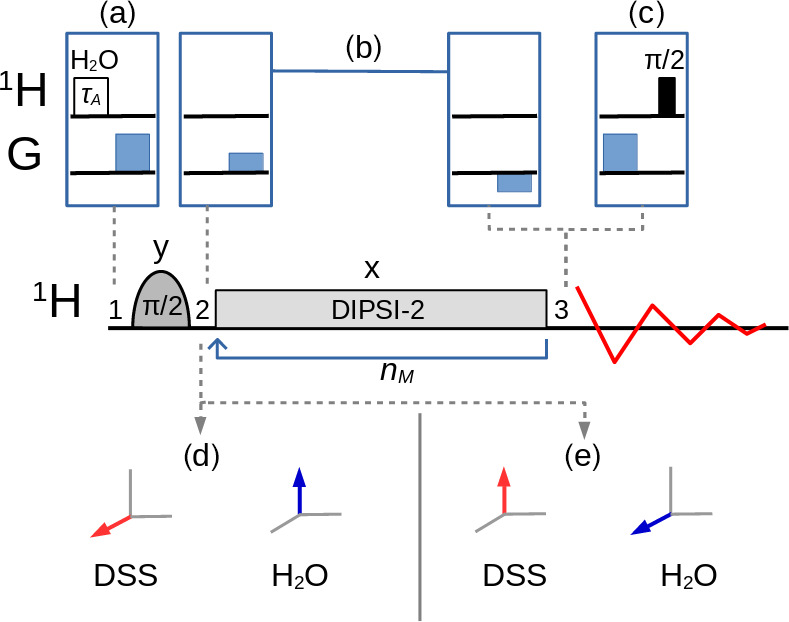
Selective TOCSY sequence. The magnetization of one nuclear spin species is rotated into the transverse plane by the selective π/2 pulse, followed by a DIPSI-2 pulse train which is repeated $n_{M}$ times. Neglecting relaxation and coherence transfer, the isotropic mixing DIPSI-2 sequence is designed to leave the magnetization unchanged (spin locked) across a wide band of frequencies centered on the RF carrier frequency. The selective pulse is cycled through ( y , -y , -y , y ) with a concomitant alternation of the receiver phase. **(a)** A selective pulse of duration $\tau_{A}$ applied to the water resonance followed by a pulsed field gradient can be inserted at position 1 to adjust the amplitude of the longitudinal components of the water magnetization between $+M_{\mathrm{eq}}^{A}$ and $-M_{\mathrm{eq}}^{A}$ . **(c)** At position 3 , a pulsed field gradient followed by a π/2 pulse permits the detection of $M_{z}$ . **(b)** An optional bipolar pulsed field gradient pair at positions 2 and 3 on each side of the mixing interval leads to a cleaner coherence pathway selection and a higher signal-to-noise ratio if the receiver gain can be increased, albeit at the cost of some signal decay due to translational diffusion (in addition, the gradient delays of about 3 ms cause a small loss due to transverse relaxation). In this work, the carrier frequency for all pulses was set either on the three methyl group resonances of DSS (leading to the situation – immediately after the selective π/2 pulse – shown in **d**) or on the water resonance (shown in **e**).

**Figure 3 Ch1.F3:**
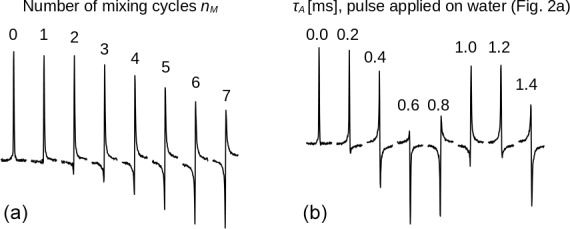
Spectra of the protons of the three methyl groups of DSS obtained with the experiment shown in Fig. [Fig Ch1.F2] with the carrier set at the methyl resonance frequency (conditions before mixing as in Fig. [Fig Ch1.F2]d). The selective π/2 pulse had a Gaussian profile of 5 ms. The strength of the RF amplitude during mixing was 4.17 kHz. **(a)** As the number of cycles $n_{M}$ increases, the signal changes phase. Each pulse train cycle takes about 7 ms to complete. After 26 cycles, the resonance is back close to its initial phase (corresponding to a precession frequency close to 5.5 Hz). **(b)** The amount of z magnetization of H ${}_{2}$ O is varied by applying a rectangular pulse with 250 Hz amplitude and a length $\tau_{A}$ (marked on top of each spectrum) to the water resonance followed by a pulsed field gradient (Fig. [Fig Ch1.F2]a) immediately before the sequence with $n_{M}=26$ . All spectra have the same phase corrections.

First, the selective TOCSY experiment shown in Fig. [Fig Ch1.F2] was applied with the RF carrier frequency set on the protons of the three methyl groups of DSS. The isotropic mixing module, DIPSI-2, consists of 36 RF pulses of constant amplitude and varying duration, applied along +x or -x , and is repeated $n_{M}$ times ([Bibr bib1.bibx21]). As the excited methyl spins S are not coupled, the mixing sequence acts as a spin lock and only a decay due to relaxation should be observed as $n_{M}$ increases. Nevertheless, the spectra shown in Fig. [Fig Ch1.F3]a display a clear phase drift, making nearly a full turn at $n_{M}=26$ . The change in phase depends strongly on the water $M_{z}^{A}$ magnetization at the beginning of the experiment, as can be seen from Fig. 3b: for $n_{M}=26$ , immediately before the selective TOCSY sequence, an RF pulse applied to the H ${}_{2}$ O resonance of varying length $\tau_{A}$ , followed by a gradient, has been inserted, so as to modify $M_{z}^{A}$ at will before the isotropic mixing sequence.

**Figure 4 Ch1.F4:**
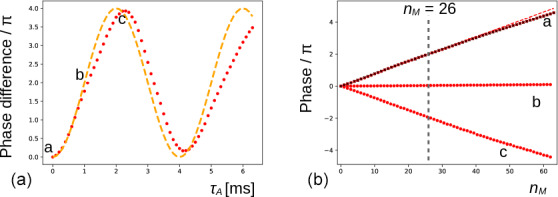
**(a)** The phase evolution of the methyl signal of DSS extracted from the experiment shown in Fig. [Fig Ch1.F3]b ( $n_{M}=26$ ). The duration of the preparatory pulse applied on H ${}_{2}$ O has been varied from 0 to 6.3 ms with increments of 0.1 ms (maximum nutation angle of ca. 3π ). The orange dashed line shows the expected variation for an ideal RF pulse. **(b)** At positions a (0 ms), b (1.1 ms, no residual solvent magnetization), and c (2.2 ms, inversion), the phase evolution of the signal is shown (red dots) for $0\leq n_{M}\leq 63$ with increments of 1. The red dashed line corresponds to a linear fit of the first 32 points of a . The black crosses are recorded under the same conditions of a after inserting a bipolar gradient pair before and after the mixing period (as explained in Fig. [Fig Ch1.F2]) and are virtually undistinguishable from the red dots underneath.

In Fig. [Fig Ch1.F4]a, the phase variations of the latter experiment are plotted as a function of $\tau_{A}$ . Clearly, the magnitude of water magnetization that is present before the pulse sequence modulates the effect observed. The theoretical curve in orange predicts the evolution of the DSS resonance assuming that the phase is proportional to the initial longitudinal water magnetization, $M_{z}^{A}$ , and the RF pulse on the water is ideal (i.e., with a nutation angle equal to $\omega_{1}\tau_{A}$ ). The deviations between the curve and the experimental points could be due to RF inhomogeneities, RD during the pulse applied to water, slight miscalibrations of the RF power, and a possible small misestimation of the initial phase shift. Moreover, as RD is a nonlinear phenomenon, it is not a priori clear that the theoretical curve should be followed. At positions a ( $\tau_{A}=0$ ms, when the water magnetization is unperturbed), b ( $\tau_{A}=1.1$ ms, when the water magnetization approximately vanishes), and c ( $\tau_{A}=2.2$ ms, when the water magnetization is approximately inverted), the phase evolution has been recorded as a function of number $n_{M}$ of isotropic mixing cycles, as shown (red dots) in Fig. [Fig Ch1.F4]b. The dashed curve corresponds to a linear regression of the first half of the points of a , showing that the dephasing slows down slightly at a larger $n_{M}$ (due to relaxation of the water magnetization). When a bipolar gradient pair is inserted to bracket the DIPSI-2 mixing sequence (black crosses), the effect of RD on the DSS resonance is almost undistinguishable from the same experiment that does not use gradients for coherence pathway selection.

In Fig. [Fig Ch1.F5]a, the three components of the magnetization of the DSS methyl groups, recorded under the same conditions as Fig. [Fig Ch1.F4] (curve a ), are plotted as a function of $n_{M}$ . Figure [Fig Ch1.F6]a shows the result of an experiment in which the carrier frequency has been moved to the solvent resonance and the amplitude of the selective Gaussian pulse has been increased in order to overcome RD effects during this pulse (so that the solvent magnetization is rotated into the xy plane). All other parameters were left unchanged. Here, the residual z component of the magnetization of DSS must be detected without changing the phase of the receiver for the different scans (the initial magnetization and the detected component have the same coherence order). Clearly, effects of the RD field are also observed in the latter experiment. Without RD, the magnetization is expected to stay along the z axis after each DIPSI-2 cycle. As in Fig. [Fig Ch1.F5], the magnetization rotates away from its initial position, although its trajectory is much less regular.

**Figure 5 Ch1.F5:**
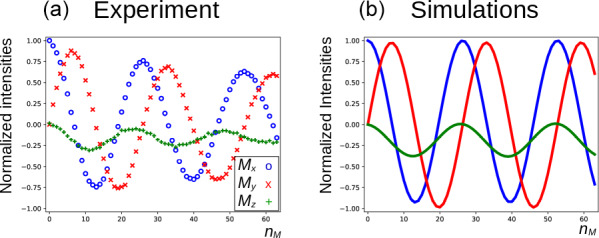
Evolution of the magnetization of the methyl resonance of DSS under the experimental conditions shown in Fig. [Fig Ch1.F4] (curve a ). For the calculations in panel **(b)**, the RD rate and angle were set to $R_{R}=33.4\times 2\pi$ rad s ${}^{-1}$ and $\psi =30{}^{\circ }$ , respectively.

**Figure 6 Ch1.F6:**
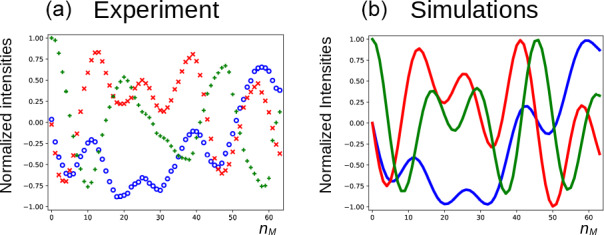
Same as Fig. [Fig Ch1.F5] but with the carrier frequency set on H ${}_{2}$ O (conditions before mixing as in Fig. [Fig Ch1.F2]e); the same symbols as in Fig. [Fig Ch1.F5] correspond to the same components of the magnetization. The RD parameters for the simulations in panel **(b)** were the same as in Fig. [Fig Ch1.F5].

## 4 Theory and discussion

In order to explain the experimental results, the homonuclear case of abundant spins A (H ${}_{2}$ O), whose magnetization induces an RD field in the coil as shown in Fig. [Fig Ch1.F1], and sparse spins S (the three methyl groups in DSS), whose RD interaction with the coil can be neglected, will be considered. In the rotating frame, the evolution of the two (uncoupled) types of spins can be described by the modified Bloch equations ([Bibr bib1.bibx5]):

1dMxi(t)dt=-ω0iMyi(t)+ω1y(t)Mzi(t)-{cRx(t)-sRy(t)}Mzi(t),2dMyi(t)dt=ω0iMxi(t)-ω1x(t)Mzi(t)-{cRy(t)+sRx(t)}Mzi(t),3dMzi(t)dt=-ω1y(t)Mxi(t)+ω1x(t)Myi(t)+{cRx(t)-sRy(t)}Mxi(t)+{cRy(t)+sRx(t)}Myi(t).

Here, i is either spin A or S , $\omega_{0}^{i}$ is the difference between the resonance frequency of spin i and the carrier frequency, $\omega_{1x}$ and $\omega_{1y}$ are the respective x and y components of the RF field during the mixing sequence, and the remaining terms in the equations are due to the RD field:

4 $$\begin{array}{r} c_{Rx}(t)=\alpha_{R}M_{x}^{A}(t)\mathrm{cos}(\psi ),\;s_{Rx}(t)=\alpha_{R}M_{x}^{A}(t)\mathrm{sin}(\psi ),\\ c_{Ry}(t)=\alpha_{R}M_{y}^{A}(t)\mathrm{cos}(\psi ),\;s_{Ry}(t)=\alpha_{R}M_{y}^{A}(t)\mathrm{sin}(\psi ), \end{array}$$

where the amplitude of the RD field is $\omega_{R}\left(t\right)=\alpha_{R}\sqrt{M_{x}^{A}{\left(t\right)}^{2}+M_{y}^{A}{\left(t\right)}^{2}}$ , and its phase is determined by the angle ψ as indicated in Fig. [Fig Ch1.F1]. The proportionality constant $\alpha_{R}$ depends on the characteristics of the RF circuit:

5 $$\alpha_{R}=\mathrm{cos}(\psi )\mu_{0}\eta \gamma Q/2,$$

where $\mu_{0}$ is the vacuum permeability, γ is the gyromagnetic ratio of the protons, η is the filling factor of the sample, and Q is the quality factor of the RF circuit. By a multiplication of $\alpha_{R}$ with the equilibrium magnetization of the abundant spins A , the use of the RD rate

6 $$R_{R}=\alpha_{R}M_{\mathrm{eq}}^{A}$$

allows one to employ normalized magnetization vectors (i.e., divide all components of spin i by $M_{\mathrm{eq}}^{i}$ ) in Eqs. ([Disp-formula Ch1.E1])–([Disp-formula Ch1.E4]).

The evolution of the magnetization of both A and S nuclei during the DIPSI-2 pulse train has been numerically simulated using the above equations. First, the evolution of $M^{A}(t)$ was determined. For spin S , Eqs. ([Disp-formula Ch1.E1])–([Disp-formula Ch1.E3]) reduce to the traditional Bloch equations, with the magnetization of spin A as a source of a time-dependent RF field. The values of the rate $R_{R}$ and the angle ψ were estimated (the two values have independently been varied in the simulations) to give a qualitative agreement with the data, as shown in Fig. [Fig Ch1.F5], rather than an exact fit. The combination of the two parameters is not unique, and a smaller angle ψ can be compensated by a larger value of $R_{R}$ . The use of the RD parameters extracted from the signal of H ${}_{2}$ O after a simple pulse-acquisition experiment does not lead to a good agreement. This is likely due to the fact that the RF circuit is not the same during signal acquisition as during the application of RF pulses ([Bibr bib1.bibx18]). In Fig. [Fig Ch1.F5], the agreement between simulations and experiments is quite satisfactory. The decay of the experimental curves is not only due to relaxation but also to RF inhomogeneities: the precession frequency of the DSS signal varies slightly with the RF amplitude, while the evolution of the z component is even more sensitive (see the Supplement). The perturbation is also present when the carrier during the mixing is set on a frequency different from DSS resonance. In the Supplement, simulations are presented for different offset frequencies.

For the curves in Fig. [Fig Ch1.F6]b, the same RD parameters as in Fig. [Fig Ch1.F5] have been used. In the simulations, the fact that, due to RD effects, the water magnetization is not aligned along the x axis after the first π/2 pulse has been taken into account (a phase shift of -18 ${}^{\circ }$ was determined experimentally). The agreement between experiments and simulations is adequate, considering the fact that neither RF inhomogeneity and calibration errors nor relaxation effects have been taken into account. Moreover, the evolution is very sensitive to the exact position of the water magnetization after the selective Gaussian pulse.

The deviation from quadrature between the RD field and the transverse magnetization of the solvent ( ψ≠0 ) plays an important role. It can be particularly pronounced in cryogenically cooled probes ([Bibr bib1.bibx24]). Previous studies have shown the influence of this “non-ideality” on the evolution of the magnetization of the abundant spins. [Bibr bib1.bibx25], [Bibr bib1.bibx31], and [Bibr bib1.bibx29] demonstrated a polarization-dependent phase shift of the freely precessing signal, while [Bibr bib1.bibx2] brought to light severe phase distortions in multiplets. [Bibr bib1.bibx32] revealed that it causes asymmetries in Z spectra, potentially perturbing the observation of chemical exchange. It is instructive to investigate what happens with a far stronger RF amplitude, which much reduces possible imperfections in the DIPSI-2 sequence due to offset effects. In Fig. [Fig Ch1.F7], several simulations of the trajectory of the DSS magnetization are presented with an amplitude of the RF field of the spin lock of 100.0 kHz. The carrier frequency was set on the water resonance, and the maximum mixing time was 0.200 s. Different initial conditions (shown above each graph) before the DIPSI-2 pulse train have been considered. When the initial magnetization of H ${}_{2}$ O is aligned along one of the principal axes, the one of DSS nutates around this axis; when this is not the case (lower right corner), the trajectory of the DSS magnetization is more complex. When the initial solvent magnetization is put along the x axis, it stays continuously almost perfectly aligned along this axis and (neglecting relaxation) the RD field is constant, with one component parallel to the magnetization equal to $\omega_{Rx}=R{}_{R}\mathrm{sin}(\psi )$ and one term perpendicular to it along the y axis equal to $\omega_{Ry}=-R_{R}\mathrm{cos}(\psi )$ . The latter component is efficiently (in analogy to the offset term) averaged out from the effective Hamiltonian by the DIPSI-2 sequence, whereas the first component is unaffected because it commutes with the RF field at all times. Hence, the effect of the RD field on the dilute spins (after each completed DIPSI-2 cycle) is expected to be a nutation around the x axis with a frequency of $R_{R}\mathrm{sin}(\psi )/2\pi$ . Indeed, this corresponds to the frequency of 16.7 Hz found in the graph on the top left corner. When the initial solvent magnetization is perpendicular (either along the z or the y axis) to the spin-lock field, the RD field becomes time dependent. The net effect is a nutation around the axis of the initial solvent magnetization with a frequency of about one-half compared with the previous one (in the Appendix, it is demonstrated that this is to be expected). When the initial magnetization of the solvent is oriented arbitrarily, the trajectory is less regular. The evolution of the solvent magnetization can also be strongly influenced by the much weaker RD field when the orientation of the initial magnetization is not along one of the three main axes (in the Supplement, the trajectories of the solvent magnetization under the same conditions as in Fig. [Fig Ch1.F7] are shown).

**Figure 7 Ch1.F7:**
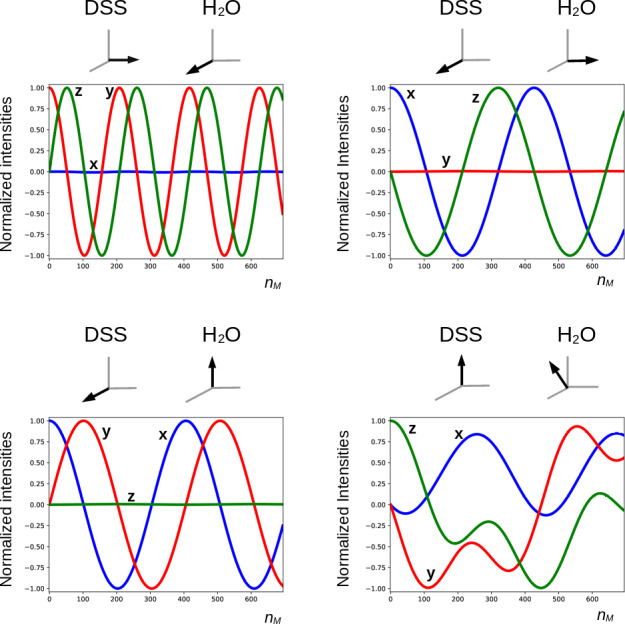
Simulated trajectories of the magnetization of the methyl groups of DSS under a DIPSI-2 pulse train with an RF amplitude of 100 kHz (90 ${}^{\circ }$ pulse of 2.5 µs ), with the carrier frequency set on the water resonance. On top of each graph, the initial magnetization just before the spin lock is shown (in the lower right corner, $M_{x}^{A}\left(0\right)/M_{\mathrm{eq}}^{A}=0.411$ , $M_{y}^{A}\left(0\right)/M_{\mathrm{eq}}^{A}=-0.310$ , $M_{z}^{A}\left(0\right)/M_{\mathrm{eq}}^{A}=0.857$ ). The RD parameters used for the simulations and the color code of the curves are identical to those used in Figs. [Fig Ch1.F5] and [Fig Ch1.F6]. For clarity, the curves are also labeled with the corresponding direction of the magnetization. The maximum number of cycles $n_{M}$ = 694 corresponds to a duration of 0.200 s.

In the previous simulations, relaxation has not been taken into account. As it causes the magnetization of the solvent to diminish, its effect on the remote resonances should become weaker as the number of spin-lock cycles increases (as the regression line in Fig. [Fig Ch1.F4] shows). In the Supplement, simulations with several different values of the transverse relaxation rates of the solvent $R_{2}^{A}$ illustrate this effect.

In this work, the selective TOCSY experiment has been investigated. For a (nonselective) two-dimensional TOCSY experiment, the situation is more complex, as, due to chemical shift evolution and RD, the orientations of the different magnetization vectors just before mixing depend on the duration of the indirect evolution period. In the Supplement, simulations are shown where the magnetization of both resonances is aligned along the x axis before the mixing period (much smaller effect) or where the magnetization of the water is aligned along the y axis while the one of DSS is along the x axis (effect comparable to Fig. [Fig Ch1.F5]).

The effect described in this article, which has been demonstrated using the DIPSI-2 mixing sequence, is expected to be present in other sequences which efficiently remove the chemical shift from the effective Hamiltonian. In the Supplement, simulations show that this is indeed the case for the MLEV-16 ([Bibr bib1.bibx16]) and the FLOPSY-16 (FLip-flOP SpectoscopY; [Bibr bib1.bibx13]) sequences. In contrast, a continuous wave spin lock with the same RF amplitude does not exhibit this effect.

The phenomenon shown in this work strongly depends on the characteristics of the probe. Similar results (not shown), albeit much smaller in magnitude, have been obtained at 18.8 T (800 MHz proton frequency) on a traditional “room temperature” probe.

## 5 Conclusions

It has been shown that, in the presence of sequences used for TOCSY mixing, RD can strongly perturb the evolution of the magnetization of spins that are neither directly coupled by scalar/dipolar interactions to the source spins nor have a nearby resonance frequency. As these types of mixing sequences efficiently remove the chemical shift differences from the effective Hamiltonian, the weak RD field affects resonances over a much wider range of frequencies than would be expected from its amplitude. Thus, counterintuitively, the RD field can cause the magnetization of remote resonances to precess notwithstanding the presence of a much stronger RF spin-locking pulse train. This effect increases with increasing RF amplitudes. Its magnitude depends on the instrumentation, the details of the pulse sequence, and the duration of the mixing time. It can be prevented by saturating or dephasing the magnetization of the spins that cause radiation damping before mixing.

## Supplement

10.5194/mr-3-43-2022-supplement The supplement related to this article is available online at: https://doi.org/10.5194/mr-3-43-2022-supplement.

## Data Availability

The data used in this study are given in the figures in this paper.

## Code availability

The code used to generate the results presented in this paper is given in the Supplement.

## Appendix

The supplement related to this article is available online at: https://doi.org/10.5194/mr-3-43-2022-supplement.

## References

[bib1.bibx1] Abragam A, Marshall WC, Wilkinson DH (1961). The international series of monographs on physics.

[bib1.bibx2] Barjat H, Chadwick G, Morris G, Swanson A (1995). The Behavior of Multiplet Signals under “Radiation Damping” Conditions. I. Classical Effects. J Magn Reson Ser A.

[bib1.bibx3] Bax A, Davis D (1985). MLEV-17-based two-dimensional homonuclear magnetization transfer spectroscopy. J Magn Reson.

[bib1.bibx4] Bloembergen N, Pound R (1954). Radiation Damping in Magnetic Resonance Experiments. Phys Rev.

[bib1.bibx5] Bloom S (1957). Effects of Radiation Damping on Spin Dynamics. J Appl Phys.

[bib1.bibx6] Braunschweiler L, Ernst R (1983). Coherence transfer by isotropic mixing: Application to proton correlation spectroscopy. J Magn Reson.

[bib1.bibx7] Broekaert P, Jeener J (1995). Suppression of Radiation Damping in NMR in Liquids by Active Electronic Feedback. J Magn Reson Ser A.

[bib1.bibx8] Chen JH, Mao XA (1997). Radiation damping transfer in nuclear magnetic resonance experiments via chemical exchange. J Chem Phys.

[bib1.bibx9] Dalvit C, Bovermann G (1995). Pulsed field gradient one-dimensional NMR selective ROE and TOCSY experiments. Magn Reson Chem.

[bib1.bibx10] Davis D, Bax A (1985). Simplification of proton NMR spectra by selective excitation of experimental subspectra. J Am Chem Soc.

[bib1.bibx11] Hobson R, Kaiser R (1975). Some effects of radiation feedback in high resolution NMR. J Magn Reson.

[bib1.bibx12] Hoult DI, Bhakar B (1997). NMR signal reception: Virtual photons and coherent spontaneous emission. Concept Magnetic Res.

[bib1.bibx13] Kadkhodaie M, Rivas O, Tan M, Mohebbi A, Shaka A (1991). Broadband homonuclear cross polarization using flip-flop spectroscopy. J Magn Reson.

[bib1.bibx14] Kessler H, Oschkinat H, Griesinger C, Bermel W (1986). Transformation of homonuclear two-dimensional NMR techniques into one-dimensional techniques using Gaussian pulses. J Magn Reson.

[bib1.bibx15] Krishnan VV, Murali N (2013). Radiation damping in modern NMR experiments: Progress and challenges. Prog Nucl Mag Res Sp.

[bib1.bibx16] Levitt M, Freeman R, Frenkiel T (1982). Broadband heteronuclear decoupling. J Magn Reson.

[bib1.bibx17] Louisjoseph A, Abergel D, Lallemand J (1995). Neutralization of Radiation Damping by Selective Feedback on a 400-Mhz Nmr Spectrometer. J Biomol NMR.

[bib1.bibx18] Marion DJ-Y, Desvaux H (2008). An alternative tuning approach to enhance NMR signals. J Magn Reson.

[bib1.bibx19] Miao XJ, Chen JH, Mao XA (1999). Selective excitation by radiation damping field for a coupled nuclear spin system. Chem Phys Lett.

[bib1.bibx20] Pöschko MT, Schlagnitweit J, Huber G, Nausner M, Hornicakova M, Desvaux H, Mueller N (2014). On the Tuning of High-Resolution NMR Probes. ChemPhysChem.

[bib1.bibx21] Rucker S, Shaka A (1989). Broadband homonuclear cross polarization in 2D N.M.R. using DIPSI-2. Mol Phys.

[bib1.bibx22] Schlagnitweit J, Morgan SW, Nausner M, Mueller N, Desvaux H (2012). Non-Linear Signal Detection Improvement by Radiation Damping in Single-Pulse NMR Spectra. ChemPhysChem.

[bib1.bibx23] Shampine L (1986). Some practical Runge-Kutta formulas. Math Comput.

[bib1.bibx24] Shishmarev D, Otting G (2011). Radiation damping on cryoprobes. J Magn Reson.

[bib1.bibx25] Sleator T, Hahn E, Hilbert C, Clarke J (1987). Nuclear-spin noise and spontaneous emission. Phys Rev B.

[bib1.bibx26] Sobol AG, Wider G, Iwai H, Wuthrich K (1998). Solvent magnetization artifacts in high-field NMR studies of macromolecular hydration. J Magn Reson.

[bib1.bibx27] Suryan G (1949). Nuclear Magnetic Resonance and the Effect of the Methods of Observation. Curr Sci.

[bib1.bibx28] Szoke A, Meiboom S (1959). Radiation Damping in Nuclear Magnetic Resonance. Phys Rev.

[bib1.bibx29] Torchia DA (2009). Slight mistuning of a cryogenic probe significantly perturbs the water H-1 precession frequency. J Biomol NMR.

[bib1.bibx30] Virtanen P, Gommers R, Oliphant TE, Haberland M, Reddy T, Cournapeau D, Burovski E, Peterson P, Weckesser W, Bright J, van der Walt SJ, Brett M, Wilson J, Millman KJ, Mayorov N, Nelson ARJ, Jones E, Kern R, Larson E, Carey CJ, Polat I, Feng Y, Moore EW, VanderPlas J, Laxalde D, Perktold J, Cimrman R, Henriksen I, Quintero EA, Harris CR, Archibald AM, Ribeiro AH, Pedregosa F, van Mulbregt P (2020). SciPy 1.0: fundamental algorithms for scientific computing in Python. Nat Methods.

[bib1.bibx31] Vlassenbroek A, Jeener J, Broekaert P (1995). Radiation damping in high resolution liquid NMR: A simulation study. J Chem Phys.

[bib1.bibx32] Williamson DC, Narvainen J, Hubbard PL, Kauppinen RA, Morris GA (2006). Effects of radiation damping on Z-spectra. J Magn Reson.

