# Supplementary material for: Radiation damping strongly perturbs remote resonances in the presence of homonuclear mixing
Source: Magn Reson (Gott). 2022 Feb 24;3(1):43–51. doi: 10.5194/mr-3-43-2022 (PMC10539763; doi:10.5194/mr-3-43-2022)
Supplement: The supplement related to this article is available online at: https://doi.org/10.5194/mr-3-43-2022-supplement. [file mr-3-43-supplement.pdf]

Supplement of Magn. Reson., 3, 43–51, 2022
https://doi.org/10.5194/mr-3-43-2022-supplement
© Author(s) 2022. CC BY 4.0 License.

Supplement of

Radiation damping strongly perturbs remote resonances in the presence of homonuclear mixing

Philippe Pelupessy

Correspondence to: Philippe Pelupessy (philippe.pelupessy@ens.psl.eu)

The copyright of individual parts of the supplement might differ from the article licence.

Contents

In this document several simulations are presented to illustrate the influence of various parameters on the RD effects during homo-nuclear total correlation mixing. The first 6 figures show simulations with parameters close to the ones in figure 5 of the main text (typically only one parameter has been varied). Figure 7 shows the trajectory of the solvent magnetization under the same conditions as in figure 7 of the main text. Finally, the Python program used for these simulations is included.

- Figure SI1) Influence of relaxation of the solvent 2
- Figure SI2) Different mixing sequences 2
- Figure SI3) RF miss-calibration 3
- Figure SI4) Variation of the RF amplitude 3
- Figure SI5) Variation of the carrier frequency ν_{RF} 4
- Figure SI6) Different initial conditions 4
- Figure SI7) Evolution of the magnetization of H_2O................ 5
- Code simulation program 6-9

Figures

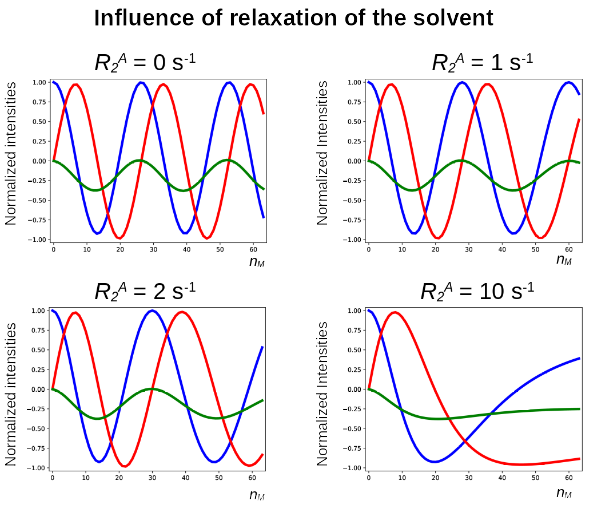

Figure SI1. Simulations of the evolution of the magnetization of the methyl resonance of DSS. Simulations with the same parameters as in figure 5 of the main article, except for the inclusion of a transverse relaxation rate R_2^A of the abundant spins A.

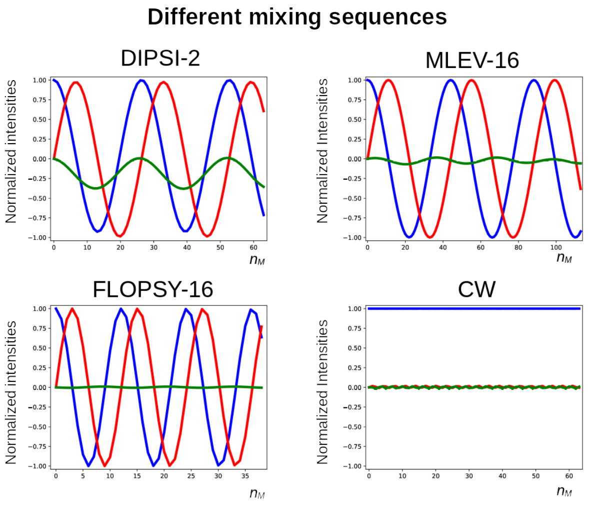

Figure SI2. Simulations of the evolution of the magnetization of the methyl resonance of DSS. Simulations of different TOCSY mixing sequences. Except for the type of mixing, all other parameters (RF and RD values) were the same as in figure 5 of the main text. The maximum number of spin-lock cycles n_M have been chosen to give approximately an equal maximum duration for the different sequences (0.435 s, 0.440 s and 0.430 s for DIPSI-2, MLEV-16 and FLOPSY-16). The continuous wave (CW) spin-lock has been simulated as the DIPSI-2 sequence but without changing the phases of the RF pulses in the pulse train.

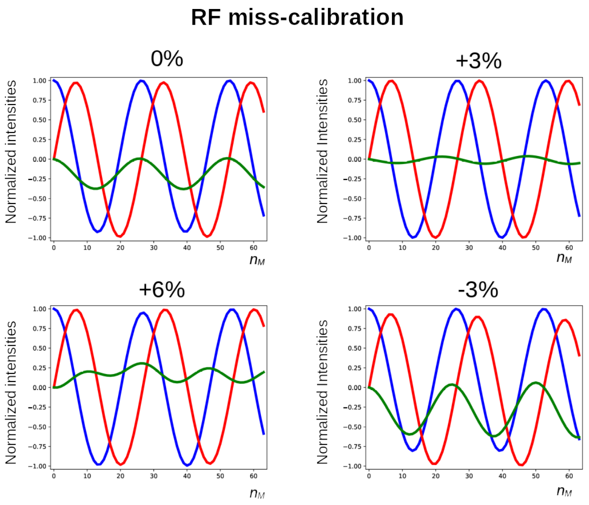

Figure SI3. Simulations of the evolution of the magnetization of the methyl resonance of DSS. Simulations as in figure 5 of the main text in order to apprehend the effects of pulse miss-calibrations and RF inhomogeneities. The RF amplitude deviates from the ideal one (4.17 kHz, corresponding to a 90° pulse of 60 μs) by a percentage indicated on top of each graph. The duration of the pulses has not been changed.

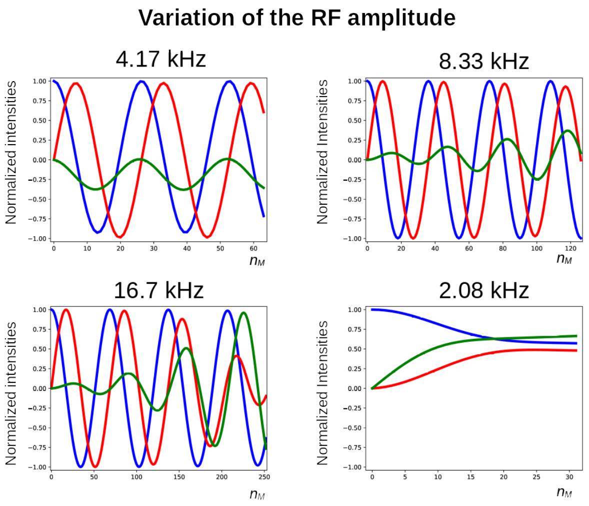

Figure SI4. Simulations of the evolution of the magnetization of the methyl resonance of DSS. Influence of the RF amplitude of the DIPSI-2 spin-lock. The different RF amplitudes are shown on top of each graph. The RF amplitudes were perfectly calibrated, hence for a doubling of the RF amplitude, the duration of the pulses must be halved. The maximum number of spin-lock cycles has been chosen as to keep the maximum spin-lock duration equal. All other parameters are identical to the ones used in figure 5 of the main text. Increasing the amplitude to 33.3 kHz (not shown) does only slightly change the aspect of the curves compared to 16.7 kHz.

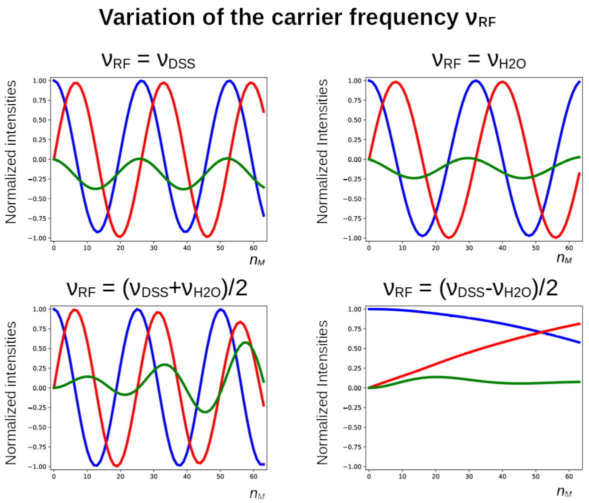

Figure SI5. Simulations of the evolution of the magnetization of the methyl resonance of DSS. The offset of the carrier has been varied (only during the mixing sequence) as shown on top of each graph. The graph where the carrier frequency is set to the methyl resonance corresponds to figure 5 in the main text. The frequencies have been set on the water resonance frequency, in the center between the water and DSS, and on the other side of the DSS resonance frequency. In the latter case the spin-lock on the water is inefficient and the effect is much weaker.

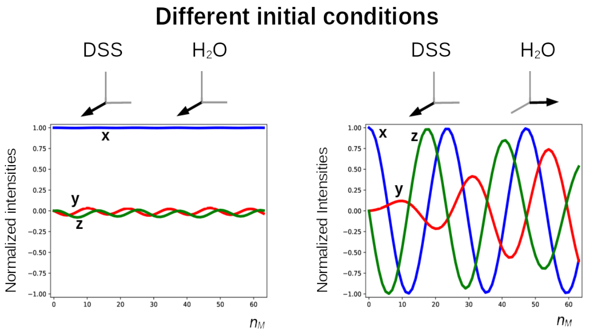

Figure SI6. Simulations of the evolution of the magnetization of the methyl resonance of DSS. The RF and RD parameters were the same as those in figure SI5 (lower left corner). (left) The magnetization of both water and DSS is aligned along the x-axis just before the mixing sequence. (right) The magnetization of water is aligned along the y-axis and the one of DSS along the x-axis. The latter graph is close to the one in figure SI5 (lower left corner) with the role of the y and z magnetization interchanged. For clarity, the curves are also labeled with the corresponding direction of the magnetization.

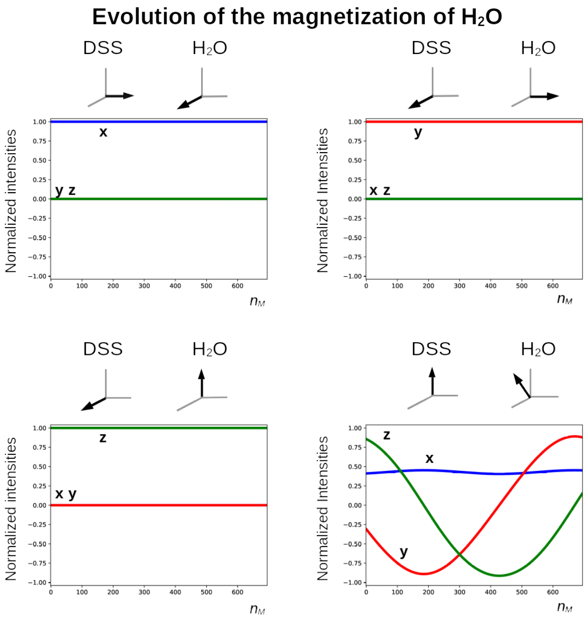

Figure SI7. Simulations of the evolution of the magnetization of the solvent resonance. The same parameters as in figure 7 of the main article have been used.

Program

```
#!/usr/bin/python

"""
nonlinBlochRD.py

Python program to calculate trajectories of magnetization in presence an RF
pulse train and radiation damping. A homonuclear system of two non-coupled
species of spins, of which only one causes radiation damping, is considered.

The program has been run with versions:

Python 3.10.1
Numpy 1.21.5
Scipy  1.7.3
Matplotlib 3.5.1

This code is provided for the purpose of checking and/or reproducing the
simulations of the publication "Radiation damping strongly perturbs remote
resonances in presence of homo-nuclear mixing" by Philippe Pelupessy in
Magnetic Resonance and the supporting information and comes without any
warranty. If you use (part of) this code for your own work, please cite the
original publication.

2022  Philippe Pelupessy
"""

# Import necessary libraries
import numpy as np
import scipy as sp
import matplotlib.pyplot as plt
from scipy.integrate import solve_ivp

def evolpassive(ini,iks,why,zed):
    """
    Rotation about an arbitrary axis: iks, why are vectors containing the
    time-dependent x and y component of the RF field and, zed is the offset
    which if we want could also be time dependent. ini is the initial density
    operator (Mx(0),My(0),Mz(0)). The output x,y,z gives the time evolution of
    the three components of the density operator.
    """
    theta = np.sqrt(iks**2+why**2+zed**2)
    a = np.cos(theta/2)
    temp = np.sin(theta/2)/theta
    b = -temp*iks
    c = -temp*why
    d = -temp*zed
    aa,bb,cc,dd = a*a, b*b,c*c, d*d
    ab,ac,ad = 2*a*b, 2*a*c, 2*a*d
    bc,bd,cd = 2*b*c, 2*b*d, 2*c*d
    x,y,z = np.zeros(len(aa)+1),np.zeros(len(aa)+1),np.zeros(len(aa)+1)
    x[0],y[0],z[0] = ini[0],ini[1],ini[2]
    for i in range(len(aa)):
        x[i+1] = ((aa[i]+bb[i]-cc[i]-dd[i])*x[i]+(bc[i]+ad[i])*y[i]+
                  (bd[i]-ac[i])*z[i])
        y[i+1] = ((bc[i]-ad[i])*x[i]+(ab[i]+cd[i])*z[i]+
                  (aa[i]-bb[i]+cc[i]-dd[i])*y[i])
        z[i+1] = ((bd[i]+ac[i])*x[i]+(cd[i]-ab[i])*y[i]+
                  (aa[i]-bb[i]-cc[i]+dd[i])*z[i])
    return x,y,z
```

```
def blochRD(t, M, wx, wy, wz, wr, a, R):
    """
    Modified bloch equations (only transverse relaxation included). The RD
    field is given by the sx, sy, cx, cy terms.
    """
    x,y,z = M
    sx = np.sin(a)*x*wr;cx = np.cos(a)*x*wr
    sy = np.sin(a)*y*wr;cy = np.cos(a)*y*wr
    return [wy*z  - wz*y - cx*z + sy*z - R*x,
            wz*x  - wx*z - cy*z - sx*z - R*y,
            -wy*x + wx*y + cx*x+cy*y -sy*x+sx*y]

def main():
    B0 = -600.1233  ## main field (minus sign for positive gyrom.)
    vH2O = 4.67     ## chemical shift H20 (ppm)
    vDSS = -0.087   ## chemical shift methyl DSS (ppm)

    mixing = 'Dipsi'
##    mixing = 'Mlev16'
##    mixing = 'Flopsy16'
##    mixing = 'CW'

## initial conditions when water is selectively excited (figure 6 of
## the article). the angles a and b account for imperfection in the
## selective pulse due to RD effects
    vrf = vH2O
    a = 0*np.pi/180;b = (-18)*np.pi/180
    iniH2O = [np.cos(a)*np.cos(b),np.cos(a)*np.sin(b),np.sin(a)]
    iniDSS = [0,0,1]

## initial conditions after selective excitation of the methyl goup of
## DSS (figure 5)
##    vrf = vDSS
##    iniH2O = [0,0,1]
##    iniDSS = [1,0,0]

## non-selective excitation
##    vrf = (vH2O)
##    iniH2O = [1,0,0]
##    iniDSS = [1,0,0]

    t90 = 60e-6 ## duration of a 90 pulse of the DIPSI-2 train
    misscal = 1.0 ## factor to account for RF amplitude errors
    RaDa = 1*210 ## RD rate
    Rangle = 1*30 ## Angle Psi

    loopmax = 64 ## maximum number of homonuclear mixing cycles
    ninc = 1 ##integer >=1, calculation done each 1/ninc degrees (1 is ok)
    rfoff = False ## if true RF  power is zero
    lockph = 0 ## add phase to spin-lock
    R2 = 0.
    if mixing == 'Dipsi':
        LockBlockPh = [0,180,0,180,0,180,0,180,0]
        LockBlockT  = [320,410,290,285,30,245,375,265,370] ##9 pulse DIPSI-2 (degr)
        SuperCycle = [0+lockph,180+lockph,180+lockph,0+lockph] ##supercycle RRbRbR

    if mixing == 'Mlev16':
        LockBlockPh = [0,90,0]
        LockBlockT = [90, 180, 90]
        SuperCycle = [0,0,180,180, 180,0,0,180, 180,180,0,0, 0,180,180,0]
```

```
if mixing == 'Flopsy16':
    LockBlockPh = [0,45,67.5,315,22.5,315,67.5,45,0]
    LockBlockT = [46,96,164,159,130,159,164,96,46]
    SuperCycle = [0,0,180,180, 180,0,0,180, 180,180,0,0, 0,180,180,0]

if mixing == 'CW':
## spin lock CW in such a way that the time increments are identical to dipsi
    LockBlockPh = [0,0,0,0,0,0,0,0,0]
    LockBlockT  = [320,410,290,285,30,245,375,265,370] ## pulse DIPSI-2 (degr)
    SuperCycle = [0,0,0,0]

tblock = 1.0*np.sum(LockBlockT)*t90/90 ## duration cycle
tsc    = tblock*len(SuperCycle) ## duration supercycle
tmax = tsc*(loopmax-1)
print('Maximum duration mixing:', np.round(tmax,3), 's')

Rangle *= np.pi/180
w1 = misscal*2*np.pi/(4*t90) ## RF power spin lock
if rfoff:w1=0
wrd = RaDa

## first calculate water magnetization
wz = B0*(vH2O-vrf)*2*np.pi  ## offset water
Mx = ([iniH2O[0]])  ## Three components water magnetization
My = ([iniH2O[1]])
Mz = ([iniH2O[2]])
rfx = ([])  ## x-component RF field
rfy = ([])  ## y-component RF field
for i in range(loopmax-1): ## loop over number spinlock cycles
    for j in range(len(SuperCycle)): ## loop supercycle
        for k in range(len(LockBlockT)): ## loop over basic elements
            ph = (np.pi/180)*(LockBlockPh[k]+SuperCycle[j]) ## phase RF
            wx = w1*np.cos(ph) ##RF amp of sequence element
            wy = w1*np.sin(ph)
            t = [0,LockBlockT[k]*t90/90] ##length element
            ini = [Mx[-1],My[-1],Mz[-1]]
            ## scipy nonlinear solver:
            sol = solve_ivp(blochRD,t,ini,args=(wx,wy,wz,wrd,Rangle,R2),
                            method='RK45',rtol =1e-12,atol =1e-9,
                            dense_output=True)
            time = np.linspace(0, LockBlockT[k]*t90/90, 1+LockBlockT[k]*ninc)
            M = sol.sol(time)
            Mx.extend(M[0][1::])
            My.extend(M[1][1::])
            Mz.extend(M[2][1::])
            rfx.extend(wx*np.ones(LockBlockT[k]*ninc))
            rfy.extend(wy*np.ones(LockBlockT[k]*ninc))
Mx,My,Mz = np.array(Mx), np.array(My), np.array(Mz)
rfx, rfy = np.array(rfx), np.array(rfy)
Mabs = np.sqrt(Mx*Mx+My*My+Mz*Mz) ##to check that the norm is conserved
plt.figure('H2O')
plt.plot(Mx[::len(SuperCycle)*np.sum(LockBlockT)*ninc],color='b',
         linewidth =4)
plt.plot(My[::len(SuperCycle)*np.sum(LockBlockT)*ninc],color='r',
         linewidth =4)
plt.plot(Mz[::len(SuperCycle)*np.sum(LockBlockT)*ninc],color='g',
         linewidth =4)
## plt.plot(Mabs[::len(SuperCycle)*np.sum(LockBlockT)*ninc])
plt.xlim([-1,loopmax])
plt.ylim([-1.04,1.04])
```

```
    ## then calculate magnetization of methyl DSS
    wz_b = B0*(vDSS-vrf)*2*np.pi ##offset

    ## the RF contains the classic RF field + RD contribution,
    ## the RF field of the total time interval is given
    wx_b = rfx+My[:-1]*RaDa*np.cos(Rangle)+Mx[:-1]*RaDa*np.sin(Rangle)
    wy_b = rfy-Mx[:-1]*RaDa*np.cos(Rangle)+My[:-1]*RaDa*np.sin(Rangle)

    dt=t90/(90*ninc)
    Mx_b,My_b,Mz_b = evolpassive(iniDSS,wx_b*dt,wy_b*dt,wz_b*dt)
    plt.figure('DSS')
    plt.plot(Mx_b[::len(SuperCycle)*np.sum(LockBlockT)*ninc],color='b',
             linewidth =4)
    plt.plot(My_b[::len(SuperCycle)*np.sum(LockBlockT)*ninc],color='r',
             linewidth =4)
    plt.plot(Mz_b[::len(SuperCycle)*np.sum(LockBlockT)*ninc],color='g',
             linewidth =4)
    plt.xlim([-1,loopmax])
    plt.ylim([-1.04,1.04])
    plt.show()

if __name__ == '__main__':
    main()
```
